# Gait Analysis in Patients After Hemiepiphysiodesis Due to Valgus or Varus Knee Deformity

**DOI:** 10.3390/jcm14020444

**Published:** 2025-01-12

**Authors:** Paweł Leyko, Monika Zaborska, Agnieszka Walczak, Łukasz Tomczyk, Marcin Pelc, Aleksander Mnich, Radosław Operacz, Piotr Morasiewicz

**Affiliations:** 1 Department of Orthopaedic and Trauma Surgery, Institute of Medical Sciences, University of Opole, 45-040 Opole, Poland; 2 Faculty of Medicine, Institute of Medical Sciences, University of Opole, 45-040 Opole, Poland; 3 University Clinical Centre in Gdańsk, 80-952 Gdańsk, Poland; 4 Department of Food Safety and Quality Management, Poznan University of Life Sciences, Wojska Polskiego 28, 60-637 Poznan, Poland; 5 Institute of Medical Sciences, University of Opole, 45-040 Opole, Poland

**Keywords:** gait, biomechanics, hemiepiphysiodesis, deformity, knee

## Abstract

**Background:** Developmental knee joint deformities are a common problem in pediatric orthopedics. Children with a valgus or varus deformity of the distal femur or the proximal tibia are commonly treated with hemiepiphysiodesis. Gait analysis in patients with lower limb deformities plays an important role in clinical practice. The purpose of our study was to assess gait parameters in patients who underwent hemiepiphysiodesis procedures of the distal femur or proximal tibia due to a knee deformity and to compare them with those in healthy controls. **Methods:** We prospectively evaluated 35 patients (14 females and 21 males) after hemiepiphysiodesis and compared the results with a healthy control group (26 participants). Gait was analyzed with a G-Sensor device (BTS Bioengineering Corp., Quincy, MA, USA). We assessed the following gait parameters: gait cycle duration, step length, support phase duration, swing phase duration, double support duration, single support duration, cadence, velocity, and step length. We assessed these gait parameters in a group of patients before and after treatment with hemiepiphysiodesis. We compared the patients’ results before and after treatment to those of a healthy control group. The level of significance was set at *p* < 0.05. **Results:** The mean follow-up period was 13 months. There was no difference in the results of gait assessments in patients prior to and after treatment. The median step length was 47.09% in the treated limb after treatment and 54.01% in the intact limb (*p* = 0.018). There were no other differences in gait parameters in the treated limbs and the healthy, intact limbs in the patient group after treatment. There were no significant differences in the patients before and after treatment compared with those in the healthy control group in all gait parameters. **Conclusions:** Valgus or varus knee deformity correction with the use of hemiepiphysiodesis does not significantly improve preoperative gait parameters. The biomechanical outcomes of hemiepiphysiodesis in the treatment of valgus or varus knee deformity are good. We observed no differences in gait cycle duration, step length, support phase duration, swing phase duration, double support duration, single support duration, gait velocity, cadence, or step length between the experimental and healthy control groups.

## 1. Introduction

Developmental knee joint deformities are a common problem in pediatric orthopedics [1,2,3,4,5,6,7,8,9,10,11,12,13,14,15,16,17,18,19]. Valgus or varus knee deformities may cause pain, physical activity limitations, limping, knee joint instability, a limited range of motion, meniscal damage, rapid joint degeneration, reduced gait efficiency, and lower limb biomechanical deficits [1,2,3,4,5,6,7,8,9,10,12,14,15,16,17,18,19]. Indications for the surgical correction of knee joint deformity are: a mechanical axis deviation (MAD) of > 1 cm or either the mechanical medial proximal tibial angle (mMPTA) or the mechanical lateral distal femoral angle (mLDFA) being increased by > 10 degrees [1,3,5,6,7,11,12,14,16,19]. Valgus deformity of the knee joint is defined as a medial shift of the center of the knee joint in relation to the mechanical axis of the lower limb, a decrease in the value of the mLDFA angle, or an increase in the value of the mMPTA angle [1,3,5,6,7,11,12,14,16,19]. Varus deformity is defined as a lateral shift of the center of the knee joint in relation to the mechanical axis of the lower limb or an increase in the mLDFA angle or a decrease in the mMPTA angle [1,3,5,6,7,11,12,14,16,19].

The conservative treatment of valgus or varus knee deformity has been shown to be ineffective [1,2,3,4,5,6,7,8,9,10,11,12,13,14,15,19]. Instead of various osteotomy techniques, children with a valgus or varus deformity of the distal femur or the proximal tibia are commonly treated with hemiepiphysiodesis [1,2,3,4,5,6,7,8,9,10,11,12,13,14,15,16,17,18,19,20,21]. In the case of large deformities (over 12 degrees) and in older children (over 14–16 years of age), osteotomies with external fixation or osteotomy with plate fixation are recommended [6,7,8,10,11,14,19]. Hemiepiphysiodesis is recommended in the case of deformities of up to 11 degrees and in children who have at least 0.5–2 years left until the predicted growth plate fusion occurs (aged 14–16 years) [6,7,8,10,11,14,19]. The effectiveness of surgical treatment in various lower-limb pathologies is generally assessed based on clinical, radiological, and biomechanical outcomes [5,7,18,22,23,24,25,26]. Abnormal biomechanical parameters after treatment may indicate persistent pain, a limited range of motion, joint instability, or joint swelling, which are indications for further treatment and rehabilitation [22,23,24,25,26]. Improved gait parameters after treatment may be the result of pain reduction, normal joint mobility, and a lack of joint instability or swelling [5,7,18,22,23,24,25,26]. Gait analysis in patients with lower limb deformities plays an important role in clinical practice, as it is essential for both evaluating the progress of rehabilitation and follow-up monitoring [22,23,26]. G-sensor devices help assess gait parameters in an objective, reproducible, and comparable way [22,24,27,28,29,30,31].

Lower limb deformities may cause pain, joint instability, limping, meniscal damage, reduced gait efficiency, and ligament laxity [1,2,3,4,5,6,7,8,9,10,12,14,15,16,17,18,19]. These knee deformity-induced abnormalities may negatively affect lower-limb biomechanics and gait parameters [5,6,7,18,22,23,24,25,26,32]. The topic of gait assessment in patients after hemiepiphysiodesis procedures has not been analyzed, with previous studies focusing mainly on clinical and radiological parameters [1,2,3,4,5,6,7,8,9,10,12,13,14,16,17,18,19,20,21].

We posited two hypotheses: (1) gait parameters would improve following hemiepiphysiodesis, and (2) gait parameters following hemiepiphysiodesis would not be worse than those in a healthy control group. The purpose of our study was to assess gait parameters in patients who underwent hemiepiphysiodesis procedures of the distal femur or proximal tibia due to a valgus or varus knee deformity and to compare them with those in healthy controls.

## 2. Materials and Methods

This study involved prospective assessments of patients prior to and after hemiepiphysiodesis, due to valgus or varus knee deformity, conducted at our clinic during the period 2023–2024. The following were our study inclusion criteria: proximal tibial or distal femoral hemiepiphysiodesis with the use of PediPlates O-Plate implants (OrthoPediatrics, Warsaw, IN, USA) due to valgus or varus knee deformity, patients aged 11–14 years, gait assessment records from before and after treatment, complete medical and radiological records over the treatment period, a follow-up period of over 6 months after treatment completion, no clinical or radiological evidence of post-treatment deformity recurrence (in still growing individuals), absence of other musculoskeletal pathologies, and consent to take part in the study. We excluded patients who: underwent no gait assessment either before or after treatment, had incomplete medical and/or radiographic records, had follow-up periods shorter than 6 months after treatment completion, had provided no consent, had other musculoskeletal disorders, or had a knee deformity of postinflammatory, metabolic, neuromuscular, or post-traumatic etiology. Our study was conducted in accordance with the Declaration of Helsinki and with the approval of the Bioethics Committee of the University of Opole (protocol code UO/0011/KB/2023, date of approval 23 March 2023). Informed consent was obtained from all subjects and/or parents/guardians involved in the study. All patients and their guardians had been informed of the voluntary nature of this study and the option of withdrawing from the study at any time. Following application of the study’s inclusion and exclusion criteria, there were 35 patients (14 females and 21 males) aged from 11 to 14 years (mean age 12 years and 5 months) remaining in the experimental group.

The control group comprised 26 healthy individuals, aged between 11 and 14 years, with no musculoskeletal pathologies. The control and experimental groups did not differ significantly in terms of mean age, height, weight, sex distribution, or BMI. The control group was identified and recruited from among children without musculoskeletal pathologies who came to the pediatric ward for diagnostic purposes and who were without any serious systemic diseases.

The patients had demonstrated clinical and radiological indications for surgical treatment. All patients included in this study underwent distal femoral or proximal tibial hemiepiphysiodesis due to a valgus or varus deformity. PediPlates O-Plate implants (OrthoPediatrics, Warsaw, IN, USA) were used in each case.

The radiological assessments were based on full weight-bearing long-standing radiographs that were obtained prior to surgery and at periodic outpatient follow-up visits. We assessed the MAD, which is defined as the perpendicular distance (expressed in millimeters) between the lower limb mechanical axis and the center of the knee joint [1,12,15,16,19,20]. We also assessed the mMPTA and the mLDFA [1,2,4,6,12,15,16,19,20,21]. Surgical correction was indicated if the MAD exceeded 10 mm or deformation (of the distal femur or proximal tibia) was > 10° or the inter-malleolar distance exceeded 8 cm, if there was radiological evidence of either no deformity reduction or deformity exacerbation after 3 months, and if the patient’s age was 1–2 years before the expected growth completion date [1,3,5,6,7,11,12,14,16,19]. The implant was placed either at the distal part of the femur or at the proximal part of the tibia, depending on the primary deformity location that was assessed based on abnormal mMPTA and mLDFA values [6,12,19]. All patients were operated on by the same two experienced orthopedic surgeons. All patients underwent the surgical procedure in a prone position under general anesthesia. Fluoroscopy was used to assess the location of the epiphyseal growth plate and for implant placement. The procedure involved making a 1–3-centimeter-long incision and positioning a PediPlates O-Plate implant under fluoroscopy to straddle the epiphyseal growth plate. On day 1 after surgery, the patients started to walk using two elbow crutches, with full weight bearing on the treated limb. All patients had the same rehabilitation protocol. Learning to walk with crutches, exercises, and physical therapy took place in the ward and up to 2 weeks after the surgery. Outpatient follow-up visits were conducted every 3 months and included lower-limb radiography. The implants were removed either after complete deformity correction or complete growth plate fusion. In those cases where complete deformity correction was achieved with the epiphyseal growth plates still present, the implant was left in until growth plate fusion or until a slight overcorrection of 3–5 degrees or MAD up to 2 mm was achieved [1,2,6,7,14,19]. In patients with growth plates present, after complete deformity correction, radiological follow-up visits were conducted every 1–2 months until a slight overcorrection was achieved. The implants were then removed, and further radiological follow-up visits were performed every 3 months until growth plate fusion.

**Figure 1:**
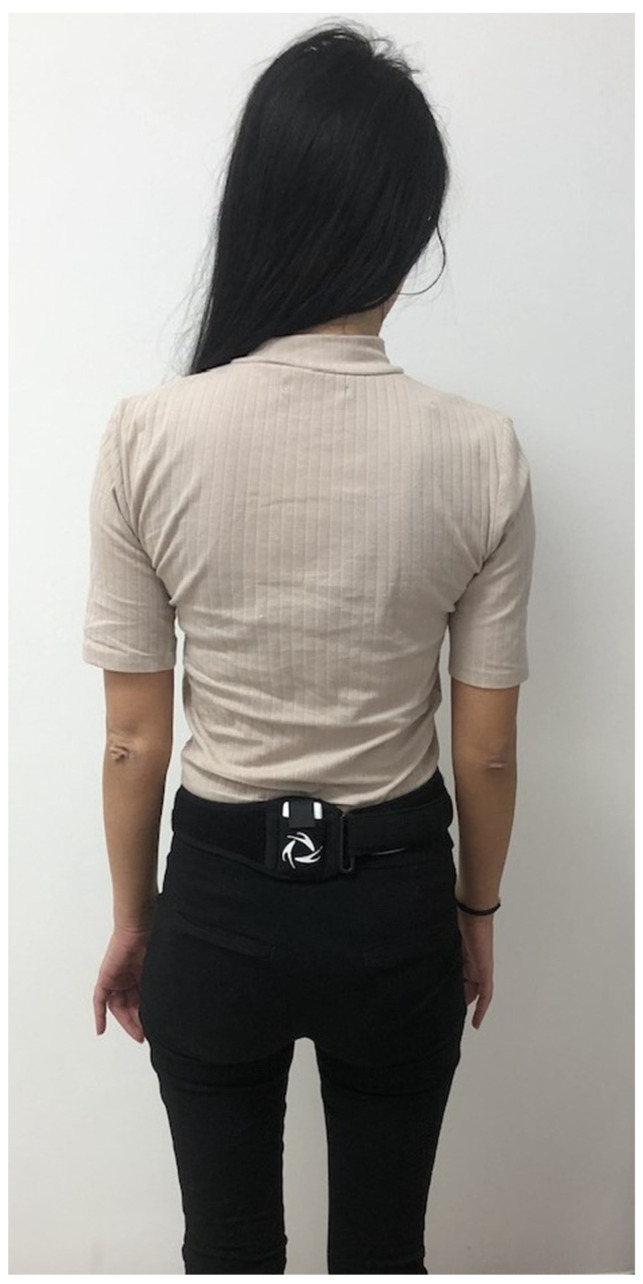
A G-sensor fitted onto a patient’s body with an elastic belt.

Gait was analyzed with a BTS G-Sensor device (BTS Bioengineering Corp., Quincy, MA, USA) equipped with an advanced triaxial accelerometer of regulated sensitivity (±2, ±4, ±8, and ±16 g), a gyroscope with regulated sensitivity (±300°/s and ±1200°/s), a triaxial magnetic field sensor, and a GPS module. A BTS G-Sensor (Figure 1) is a lightweight (37 g), compact device measuring 70 × 40 × 18 mm (length × width × height) that ensures precise measurements. The sampling frequency was up to 100 Hz, and the data were sent to a computer via a Bluetooth wireless transfer and then processed with the designated BTS G-Studio software. The software package provided by the manufacturer helped us calculate the spatiotemporal parameters of their gait and the percentage of symmetry between the two limbs.

**Figure 2:**
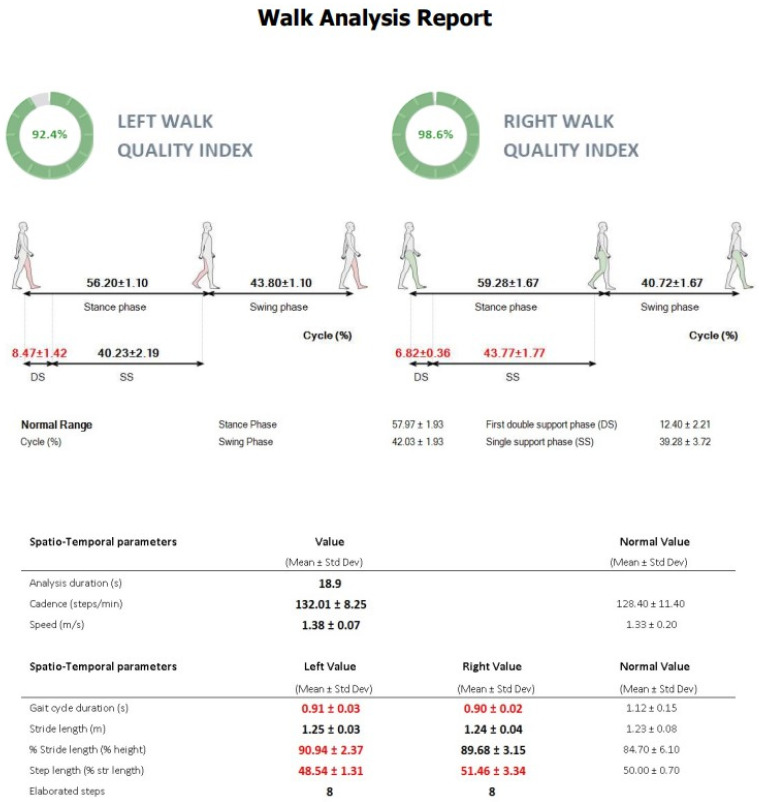
An example report of gait analysis with the G-Sensor device.

Each patient was provided with detailed instructions on the measurement procedure. All study participants had their body weight and height measured. Subsequently, everyone was fitted with a wireless BTS G-Sensor secured by a semi-elastic belt at the level of the fifth lumbar vertebra (L5), as shown in Figure 1. A G-Sensor device is suitable for gait parameter assessment, as indicated by an inter-instrument coefficient of variation of 2.5% and analyses of the inter-instrument correlation coefficient from 0.90 to 0.99 [30,31]. A G-sensor ensures an objective, accurate, and reproducible assessment of all phases of gait, as well as the detection of any gait abnormalities [22,24,27,28,29,30,31]. The assessment was conducted with the use of the WALK protocol, beginning with a short period of the person remaining motionless to allow the sensor to automatically stabilize, and subsequently walking for 8 m in a straight line in one direction, turning around, and walking back to the starting point. Study participants walked barefoot at their standard walking speed. All assessments for the patient group and control group were performed in the same room and by the same investigator (one experienced orthopedist). Each patient and participant from the control group underwent three measurements, with their mean values used in further analyses. The accrued data were recorded with dedicated BTS G-Studio software, which helps process the data and calculate the spatiotemporal parameters of gait and the percentage of symmetry between the two limbs, as shown in Figure 2. Subsequently, the results were exported into a spreadsheet and analyzed statistically. We assessed and analyzed the following gait parameters:

- Gait cycle duration (s);
- Step length (%);
- Support phase duration (%);
- Swing phase duration (%);
- Double support duration (%);
- Single support duration (%);
- Cadence (steps/min);
- Velocity (m/s);
- Step length (m).

The median step length (m) was calculated for both limbs (the healthy and treated limbs for the patient group and the dominant and non-dominant limbs for the control group).

Gait analyses were performed in patients 1–3 months before surgery and at least 6 months after implant removal (6–16 months; on average, 13 months). The post-treatment gait assessment results of patients were compared with the gait assessment results for the healthy control group. As previously reported in the literature, the dominant lower limbs analyzed in the control group were compared with the healthy limbs in the experimental group, and the non-dominant lower limbs in the control group were compared with the treated limbs in the experimental group [32,33,34].

### Statistical Analysis

Data were statistically analyzed using Statistica 13.3. The Shapiro–Wilk test was used to check for normality of distribution. The Mann–Whitney U test was used to compare quantitative variables. The level of statistical significance was set at *p* < 0.05.

The sample size of the present study was calculated by averaging the prevalence of detailed gait parameters from studies of similar cohorts. We estimated the average prevalence of the present study to be around 10% in order to achieve a 95% confidence interval with 5% precision; thus, we required a sample size of at least 20 participants.

## 3. Results

The mean follow-up period was 13 months (ranging from 6 to 16 months). The results of the gait assessments of patients prior to and after treatment are presented in Table 1. The median gait velocity in patients was 1.07 m/s prior to treatment and 1.07 m/s at post-treatment follow-up (*p* = 0.863, Table 1). The median cadence in patients was 104.45 steps/min prior to treatment and 102.04 steps/min after treatment; the difference was not statistically significant (*p* = 0.378, Table 1).

The median step length in patients was 1.125 m prior to treatment and 1.27 m at post-treatment follow-up; the difference between these values was not statistically significant (*p* = 0.378, Table 1).

The median post-treatment gait velocity in patients was 1.07 m/s (at follow-up), and the median gait velocity in the control group of healthy volunteers was 1.065 m/s; these differences were not statistically significant (*p* = 0.404, Table 2). We observed no statistically significant differences between the median cadence in post-treatment patients (102.04 steps/min) and that in healthy controls (107.855 steps/min) (*p* = 0.17, Table 2). The median step length was 1.27 m in post-treatment patients and 1.11 m in healthy volunteers; this difference was not statistically significant (*p* = 0.099, Table 2).

A detailed comparison of gait parameters in patients before treatment and in the healthy control group is presented in Table 3. There were no significant differences in the patients before treatment compared with those in the healthy control group for the following median gait parameters: gait cycle duration, step length, support phase duration, swing phase duration, double support duration, or single support duration (Table 3).

**Figure 3:**
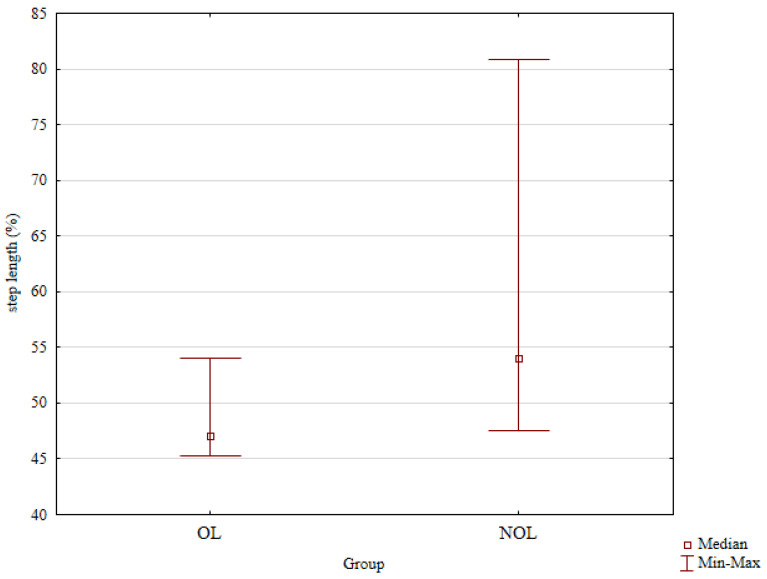
The post-treatment median step length in the treated limbs and the healthy limbs.

A detailed comparison of the gait parameters in the treated and the healthy, intact limbs of patients after treatment is presented in Table 4. The median gait cycle duration was 1.16 s in the treated limb and 1.17 s in the healthy limb; these differences were not statistically significant (Table 4). The median step length was 47.09% in the treated limb after treatment and 54.01% in the intact limb; this difference was statistically significant (*p* = 0.018, Figure 3, and Table 4).

In patients after treatment, the median support phase duration was 59.36% in the treated limb and 59.78% in the intact, healthy limb; this difference was not significant (*p* = 0.665, Table 4). Moreover, there was no significant difference in the median swing phase duration between the treated limb (40.91%) and intact limb (40.22%) in patients (*p* = 0.36, Table 4). At post-treatment follow-up, the median double support duration was 8.99% in the treated limb and 9.67% in the healthy limb; the difference was not significant (*p* = 0.531, Table 4). There was no significant difference in median single support duration (40.46%) between the treated limb after treatment and the intact limb (41.6%); *p* = 0.47, Table 4.

A detailed analysis of the various gait parameters between patients after treatment and the control individuals is presented in Table 5. There were no significant differences in the patients after treatment compared with those in the healthy control group for the following median gait parameters: gait cycle duration, step length, support phase duration, swing phase duration, double support duration, or single support duration (Table 5).

## 4. Discussion

There are no publications in the available literature that assess the gait of patients after hemiepiphysiodesis. The purpose of this study was to prospectively assess the effects of using hemiepiphysiodesis for valgus or varus knee deformity correction on gait parameters and to compare the gait parameters of patients with those of healthy individuals. Our study results showed no significant differences in the assessed gait parameters before and after the treatment of valgus or varus knee deformity with the use of hemiepiphysiodesis. The results for these gait parameters assessed before and after treatment in patients were not significantly different from those in the healthy control group. The results of our study partly support our research hypotheses.

As postulated by other authors, lower limb deformities, including valgus and varus knee deformities, may cause pain, joint instability, limping, meniscal damage, reduced gait efficiency, and ligament laxity [1,2,3,4,5,6,7,8,9,10,12,14,15,16,17,18,19,35]. These knee-deformity-induced abnormalities may negatively affect lower limb biomechanics and gait parameters [5,6,7,18,22,23,24,25,26,32,35]. Hemiepiphysiodesis is used to correct valgus and varus knee deformities in children [1,2,3,4,5,6,7,8,9,10,11,12,13,14,15,16,17,18,19,20,21], which, theoretically, should benefit their musculoskeletal biomechanics and improve their gait parameters. Deformity correction, achieving a normal range of motion, and postoperative pain reduction are key to the process of regaining full mobility and restoring normal patterns of motion after treatment [22,23].

Other authors have focused on assessing clinical and radiological parameters after using hemiepiphysiodesis to treat knee joint deformities in children [1,2,3,4,5,6,7,8,9,10,12,13,14,16,17,18,19,20,21]. Despite some authors reporting that valgus or varus knee deformities may cause gait abnormalities [2,3,4,5,6,7,9,14,17,18], there are no available reports of studies assessing the effects of valgus and varus knee deformities on gait parameters in children and the changes in these parameters after treatment with the use of hemiepiphysiodesis. Apart from the clinical and radiological parameters, assessing the biomechanics and gait parameters is important from the perspectives of orthopedic surgeons, rehabilitants, and patients [5,7,18,22,23,24,25,26,32,35].

The goal of hemiepiphysiodesis is deformity correction, which may be crucial for the normal biomechanics of gait [2,3,4,5,6,7,9,14,17,18,24]. Goldman and Green reported that knee joint deformities may alter the biomechanics of gait by shifting the mechanical axis and altering the joint load; they may also cause pain, knee joint instability, and ligament laxity [5]. According to other authors, valgus and varus knee deformities may cause pain and ligament laxity, which may lead to limping and other gait abnormalities [7,18]. As shown by our study, knee deformity correction did not affect the gait parameters in any significant way, which may be due to several reasons. First, the knee joint deformities in our patient population did not cause any pain, range-of-motion limitations, muscle weakness, or joint instability before treatment. Second, epiphysiodesis is indicated in minor and moderate deformities (major deformities require osteotomy), which may not cause joint instability, ligament laxity, or pain. Third, the age of patients in our cohort (mean age: 12 years and 5 months) ensured greater compensatory and adaptation capacity of the musculoskeletal system (such as high overall fitness levels, unrestricted range of motion, and proportionally strong muscles) in comparison with that of adults, which helped eliminate the negative effects of moderate and minor deformities on the mechanism of gait. In the group of patients we assessed, the mean amount of MAD correction was 16.9 mm. Our study showed that the use of hemiepiphysiodesis for the correction of minor and moderate valgus or varus deformities of the knee joint does not significantly affect changes in gait parameters and lower limb biomechanics. Minor and moderate deformities of the knee joint do not change the gait parameters, which may result from the lack of pain, instability, or limited movement in these patient groups.

A G-sensor evaluation of a group of 22 patients after symptomatic pes planovalgus treatment with a Spherus talar screw showed significant differences in post-treatment gait parameters of the treated and intact limb only in terms of step length [22]. Those authors observed no differences in the duration of the single support phase, double support phase, swing phase, support phase, or gait cycle [22]. Neither were there any significant differences in the preoperative duration of the single support phase, double support phase, swing phase, support phase, or gait cycle [22]. Salas-Gomez et al. used a G-Sensor to assess the gait of 22 adult patients who had suffered bimalleolar ankle fractures [24]. Those authors noted a significant difference in single support duration in the treated and intact limbs (32.6% and 36.7%, respectively) [24]. They also noted significant differences between the treated and intact limbs in terms of swing phase duration, support phase duration, and step length [24]. Other authors assessed gait parameters in 50 patients aged 3.5–6 years with primitive reflexes [28]. Those authors found that reflex activity affects the following gait parameters: the left step length, the right single support phase, the right double support phase, and the left double support phase [28]. The measurements obtained in the gait analysis conducted for our study were comparable with those obtained by other authors [22,24,28], which indicates the reproducibility of those measurements.

Our detailed post-treatment assessment of gait demonstrated significant differences in the patients’ treated and intact limbs for a single parameter—step length. These results show that hemiepiphysiodesis had no negative effect on gait parameters and lower limb biomechanics after treatment.

We did not observe any significant differences between the patients (post-treatment) and the healthy controls in terms of the median values of the following parameters: gait cycle duration, step length, support phase duration, swing phase duration, double support duration, and single support duration. These parameters showed no differences for either the treated limbs or the untreated limbs of patients, in comparison with the non-dominant and dominant limbs of the controls, respectively. Therefore, we can assume that valgus or varus knee deformity treatment with the use of hemiepiphysiodesis helps achieve gait parameters comparable with those of healthy individuals.

Bobiński et al. used a G-sensor to assess the gait in 22 patients, aged 7–14 years, after symptomatic pes planovalgus treatment with a Spherus talar screw [22]. Those authors observed no post-treatment differences between the treated and intact limb in the duration of the single support phase, double support phase, gait cycle, swing phase, or single support [22]. The mean post-treatment cadence was 102.94 steps/min, the mean gait velocity was 0.96 m/s, and the mean step length was 0.57 m [22]. Those authors noted postoperative shortening of the gait cycle duration in the operated limb to 1.24 s [22]. Another study assessed gait parameters in 22 adult patients (mean age 43.5 years) 6 months after bimalleolar ankle fractures [24]. In this group, the mean gait velocity was 0.94 m/s, the mean cadence was 99.9 steps/min, and the mean step length was 1.28 m [24]. There were differences between the intact and treated limbs in terms of stance phase, swing phase, single support phase duration, and step length [24]. Moreover, there were differences between the patient and control groups in terms of gait velocity, cadence, and stride length [24]. Gaebe and his team assessed gait in 12 patients with pseudoachondroplasia, with valgus or varus deformity of the knee [35]. The cohort had large deformities (from 29 degrees valgus to 48 degrees varus deformities). The average cadence in the assessed group was 127 steps/min and the average speed was 87.1 cm/s [35]. Gieysztor et al. used a G-sensor to assess the gait of 50 patients aged 3.5–6 years with primitive reflexes [28]. In this group of patients, the mean values of cadence, gait velocity, and step length were 136.6 steps/min, 0.8 m/s, and 0.7 m, respectively [28]. The values of gait velocity, cadence, and step length measured in the patients in our study were similar to those reported by other authors [22,24,28,35].

An earlier study involved the use of a G-sensor to assess gait parameters in healthy children [27]. The mean gait velocity, cadence, and duration of the single support phase, double support phase, and swing phase results reported by Gieysztor et al. [27] were comparable with the respective post-treatment results in our patients. This indicates no changes in gait parameters following valgus or varus knee deformity treatment with the use of hemiepiphysiodesis. Gait parameters in normally developing children are expected to be symmetrical, which indicates normal lower-limb biomechanics [27,29]. In our study, the experimental group with knee joint deformity showed most of the post-treatment gait parameters (except the step length in the treated and intact, healthy limbs) to be symmetrical, which suggests good treatment outcomes.

One factor that may affect the outcomes of using hemiepiphysiodesis in knee joint deformity treatment is the deformity etiology [1,4,5,7,11]. Therefore, we excluded deformities due to trauma, inflammation, neurogenic factors, and rickets from our study. We assessed only idiopathic knee deformities. According to some authors, the type of knee joint deformity (valgus vs. varus) does not affect the outcomes of treatment with hemiepiphysiodesis [20,21].

One limitation of our study is the relatively small sample size. This is due to the prospective nature of our study and our decision to use relatively numerous and strict exclusion criteria in order to obtain a homogeneous study group. Other authors have also compared populations of a similar size to, or even smaller than, ours [3,8,9,10,13,18,20,22,24,25,35].

One strength of our study was the use of the same operative and rehabilitation protocol in all patients, having all surgeries conducted by the same two experienced orthopedic surgeons, and having all gait assessments be conducted by one doctor. Another strength of our study is its prospective design and the fact that we compared our gait assessment results with the ones obtained for a healthy control group. Still another strength of our study is the fact that the only patients that we assessed were the ones with idiopathic knee deformities and that all evaluated patients were within a narrow age range of 11–14 years. In the future, we are planning a similar study in a larger patient population, with a longer follow-up. We plan to perform similar gait assessment tests, dividing patients into a group with the correction of valgus deformity and a group with the correction of varus deformity.

## 5. Conclusions

Valgus or varus knee deformity correction with the use of hemiepiphysiodesis does not significantly improve preoperative gait parameters. The biomechanical outcomes of hemiepiphysiodesis in the treatment of valgus or varus knee deformity are good. We observed no differences in gait cycle duration, step length, support phase duration, swing phase duration, double support duration, single support duration, gait velocity, cadence, or step length between our experimental and healthy control groups.

## Figures and Tables

**Table 1:** Selected gait parameters before and after surgery in the patient group.

Analyzed Variable		Before Surgery	After Surgery	Z	*p*
cadence (steps/min)	Q1	98.82	96.33	−0.879	0.378
	Median	104.45	102.04		
	Q3	119.02	108.14		
velocity (m/s)	Q1	0.89	0.95	−0.171	0.863
	Median	1.07	1.07		
	Q3	1.16	1.2		
step length (m)	Q1	1.01	1.19	−0.879	0.378
	Median	1.13	1.27		
	Q3	1.26	1.38		
analysis duration (s)	Q1	19.45	16.5	−0.525	0.599
	Median	21.7	18.9		
	Q3	28.45	23.9		

Z—standardized value of the Mann–Whitney U test; *p*—value for the Mann–Whitney U test; Q1, Q3—1st and 3rd quartiles.

**Table 2:** Selected gait parameters in the Patients group after treatment and the healthy Control group.

Analyzed Variable		Group		Z	*p*
		Patients	Control		
analysis duration (s)	Q1	16.5	20.8	−1.528	0.126
	Median	18.9	26.5		
	Q3	23.9	28.4		
cadence (steps/min)	Q1	96.33	100.25	−1.369	0.17
	Median	102.04	107.86		
	Q3	108.14	112.37		
velocity (m/s)	Q1	0.95	0.81	0.833	0.404
	Median	1.07	1.07		
	Q3	1.2	1.11		
step length (m)	Q1	1.19	0.92	1.647	0.099
	Median	1.27	1.11		
	Q3	1.38	1.28		

Z—standardized value of the Mann–Whitney U test; *p*—value for the Mann–Whitney U test; Q1, Q3—1st and 3rd quartiles.

**Table 3:** Detailed gait parameters in the Patients group before treatment and the healthy Control group.

Analyzed Variable		Group		Z	*p*
		Patients	Control		
gait cycle duration OL (s)	Q1	0.96	1.1	−0.416	0.678
	Median	1.11	1.13		
	Q3	1.22	1.21		
step length OL (%)	Q1	45.9	47.75	−1.642	0.101
	Median	48.03	49.1		
	Q3	49.49	50.15		
support phase duration OL (%)	Q1	58.12	57.9	−0.021	0.983
	Median	59.85	60.39		
	Q3	61.71	61.84		
swing phase duration OL (%)	Q1	38.29	38.16	0.021	0.983
	Median	40.14	39.61		
	Q3	41.87	42.1		
double support duration OL (%)	Q1	7.96	8.46	−1.309	0.190
	Median	9.08	11.71		
	Q3	10.21	12.17		
single support duration OL (%)	Q1	39.4	37.11	2.037	0.042
	Median	40.33	38.21		
	Q3	41.34	40.63		
steps analyzed OL	Q1	4.5	4	−1.600	0.109
	Median	5	7.5		
	Q3	6	8		
gait cycle duration NOL (s)	Q1	0.98	1.09	−0.375	0.708
	Median	1.11	1.13		
	Q3	1.21	1.2		
step length NOL (%)	Q1	50.1	49.85	1.559	0.119
	Median	51.97	50.89		
	Q3	54.1	52.25		
support phase duration NOL (%)	Q1	58.05	60.11	−1.912	0.056
	Median	59.21	61.1		
	Q3	60.67	62.6		
swing phase duration NOL (%)	Q1	39.33	37.4	1.912	0.056
	Median	40.78	38.89		
	Q3	41.95	39.89		
double support duration NOL (%)	Q1	9.49	7.35	−0.333	0.739
	Median	10.37	10.72		
	Q3	11.04	12.5		
single support duration NOL (%)	Q1	38.7	37.5	0.520	0.603
	Median	39.71	39.31		
	Q3	42.05	41.62		
steps analyzed NOL	Q1	4	4	−1.446	0.148
	Median	5	7		
	Q3	6	8		

OL—operated limb, NOL—non-operated limb; Z—standardized value of the Mann–Whitney U test; *p*—value for the Mann–Whitney U test; Q1, Q3—1st and 3rd quartiles.

**Table 4:** Spatiotemporal parameters of gait in the Patients group after treatment.

Analyzed Variable		OL	NOL	Z	*p*
		Median ± Standard Deviation			
gait cycle duration (s)	Q1	1.1	1.145	−0.433	0.665
	Median	1.16	1.17		
	Q3	1.27	1.23		
step length (%)	Q1	46.69	48.72	−2.357	0.018
	Median	47.09	54.01		
	Q3	50.76	54.635		
support phase duration (%)	Q1	57.11	59.015	−0.433	0.665
	Median	59.36	59.78		
	Q3	62.14	60.64		
swing phase duration (%)	Q1	37.96	39.36	0.914	0.36
	Median	40.91	40.22		
	Q3	42.89	40.985		
double support duration (%)	Q1	8.3	8.545	−0.625	0.531
	Median	8.99	9.67		
	Q3	10	12.19		
single support duration (%)	Q1	38.85	39.725	−0.721	0.47
	Median	40.46	41.60		
	Q3	41.27	41.925		
steps analyzed	Q1	4.00	3	1.299	0.193
	Median	4.00	3.50		
	Q3	4.00	4		

OL—operated limb, NOL—non-operated limb; Z—standardized value of the Mann–Whitney U test; *p*—value for the Mann–Whitney U test; Q1, Q3—1st and 3rd quartiles.

**Table 5:** Detailed gait parameters in the Patients group after treatment and the healthy Control group.

Analyzed Variable		Group		Z	*p*
		Patients	Control		
gait cycle duration OL (s)	Q1	1.1	1.1	0.377	0.705
	Median	1.16	1.14		
	Q3	1.27	1.21		
step length OL (%)	Q1	46.69	47.75	−1.102	0.27
	Median	47.09	49.11		
	Q3	50.76	50.15		
support phase duration OL (%)	Q1	57.11	57.9	0.094	0.924
	Median	59.36	60.39		
	Q3	62.14	61.84		
swing phase duration OL (%)	Q1	37.96	38.16	0.22	0.825
	Median	40.91	39.61		
	Q3	42.89	42.1		
double support duration OL (%)	Q1	8.3	8.46	−1.228	0.219
	Median	8.99	11.71		
	Q3	10	12.17		
single support duration OL (%)	Q1	38.85	37.11	1.795	0.072
	Median	40.46	38.21		
	Q3	41.27	40.63		
steps analyzed OL	Q1	4	4	−1.7	0.088
	Median	4	7.5		
	Q3	4	8		
gait cycle duration NOL (s)	Q1	1.15	1.09	1.092	0.274
	Median	1.17	1.13		
	Q3	1.23	1.2		
step length NOL (%)	Q1	48.72	49.85	0.989	0.322
	Median	54.01	50.89		
	Q3	54.64	52.25		
support phase duration NOL (%)	Q1	59.02	60.11	−1.057	0.29
	Median	59.78	61.11		
	Q3	60.64	62.6		
swing phase duration NOL (%)	Q1	39.36	37.4	1.057	0.29
	Median	40.22	38.89		
	Q3	40.99	39.89		
double support duration NOL (%)	Q1	8.55	7.35	−0.238	0.811
	Median	9.67	10.72		
	Q3	12.19	12.5		
single support duration NOL (%)	Q1	39.73	37.5	0.989	0.322
	Median	41.59	39.32		
	Q3	41.93	41.62		
steps analyzed NOL	Q1	3	4	−2.968	0.059
	Median	3.5	7		
	Q3	4.00	8		

Z—standardized value of the Mann–Whitney U test; *p*—value for the Mann–Whitney U test; Q1, Q3—1st and 3rd quartiles.

## Data Availability

The data presented in this study are available on request from the corresponding author.
